# Selective Preservation of Bone Marrow Mature Recirculating but Not Marginal Zone B Cells in Murine Models of Chronic Inflammation

**DOI:** 10.1371/journal.pone.0011262

**Published:** 2010-06-22

**Authors:** Elisabetta Traggiai, Anna Casati, Michela Frascoli, Simona Porcellini, Maurilio Ponzoni, Francesca Sanvito, Lin Leng, Richard Bucala, Lorenzo Moretta, Fabio Grassi

**Affiliations:** 1 Istituto Giannina Gaslini, Genoa, Italy; 2 Institute for Research in Biomedicine, Bellinzona, Switzerland; 3 Department of Biology and Genetics for Medical Sciences, University of Milan, Milan, Italy; 4 Pathology Unit, San Raffaele Scientific Hospital, Milan, Italy; 5 Department of Medicine, Yale University School of Medicine, New Haven, Connecticut, United States of America; 6 University of Genoa, Genoa, Italy; University Paris Sud, France

## Abstract

Inflammation promotes granulopoiesis over B lymphopoiesis in the bone marrow (BM). We studied B cell homeostasis in two murine models of T cell mediated chronic inflammation, namely calreticulin-deficient fetal liver chimeras (FLC), which develop severe blepharitis and alopecia due to T cell hyper responsiveness, and inflammatory bowel disease (IBD) caused by injection of CD4^+^ naïve T cells into lymphopenic mice. We show herein that despite the severe depletion of B cell progenitors during chronic, peripheral T cell-mediated inflammation, the population of BM mature recirculating B cells is unaffected. These B cells are poised to differentiate to plasma cells in response to blood borne pathogens, in an analogous fashion to non-recirculating marginal zone (MZ) B cells in the spleen. MZ B cells nevertheless differentiate more efficiently to plasma cells upon polyclonal stimulation by Toll-like receptor (TLR) ligands, and are depleted during chronic T cell mediated inflammation in vivo. The preservation of mature B cells in the BM is associated with increased concentration of macrophage migration inhibitory factor (MIF) in serum and BM plasma. MIF produced by perivascular dendritic cells (DC) in the BM provides a crucial survival signal for recirculating B cells, and mice treated with a MIF inhibitor during inflammation showed significantly reduced mature B cells in the BM. These data indicate that MIF secretion by perivascular DC may promote the survival of the recirculating B cell pool to ensure responsiveness to blood borne microbes despite loss of the MZ B cell pool that accompanies depressed lymphopoiesis during inflammation.

## Introduction

An ordered sequence of phenotypes characterizes B cell development in the BM from the common lymphoid progenitor (CLP) (Lin^−^c-Kit^lo^IL-7R^+^) [1]. Rearrangement of the IgH locus determines the expression of the pre-B cell receptor (pre-BCR) and the transition of pro-B cells to the pre-B cell (B220^lo^CD2^+^) stage [2]. The progression to the following immature B cell stage (B220^+^IgM^+^IgD^lo^) is characterized by expression of the BCR. Immature B cells leave the BM and develop into mature follicular (FO) B cells (IgM^+/lo^IgD^+^) in the spleen. These cells recirculate and home to B cell areas in secondary lymphoid organs where they mediate T-dependent humoral immune responses. A distinct non-recirculating mature population in the spleen is constituted by MZ B cells (IgM^hi^IgD^lo^), which line the marginal sinus and respond to blood-borne antigens. Together with peritoneal and mucosal B-1 B cells MZ B cells provide T-independent immune responsiveness to multivalent antigens and generate the natural antibody repertoire [3]. Recently, a subset of recirculating mature B cells was shown to occupy a perisinusoidal niche in the BM and to be activated by blood-borne pathogens in a T cell-independent fashion to generate specific IgM responses, analogously to MZ B cells [4].

The increased peripheral demand of granulocytes during an infection or inflammatory condition promotes granulopoiesis over B lymphopoiesis in the BM [5], [6]. The consequences of inflammation on B cell subsets representation have not been investigated to date. We used two murine models of T cell mediated chronic inflammation to address this issue. Calreticulin-deficient (*crt^−/−^*) T cells display an altered regulation of calcium signalling upon TCR stimulation, which results in protracted nuclear translocation of nuclear factor of activated T cell (NFAT) and activation of the mitogen activated protein kinase (MAPK) pathway. This results in lower threshold of T cell activation and hyper responsiveness upon antigen encounter. Adoptive transfer of *crt^−/−^* fetal liver progenitors into recombinase-deficient mice to reconstitute the lymphoid system determines a T cell dependent inflammatory disease characterized by severe blepharitis and alopecia [7]. As a second model of chronic inflammation we used *cd3e^−/−^* mice in which we induced IBD by transfer of naïve CD4^+^ T cells [8]. We demonstrate that chronic inflammation results in MZ but not BM mature B cells depletion. MIF, which constitutes a crucial survival signal for BM mature B cells [9], was increased during inflammation. Pharmacological inhibition of MIF determined reduction of BM mature B cells, thereby suggesting a function for MIF in ensuring blood borne antigens responsiveness upon depletion of MZ B cells.

## Results

### Altered BM myeloid and lymphoid lineage representation in mice with T cell mediated inflammation

**Figure 1:**
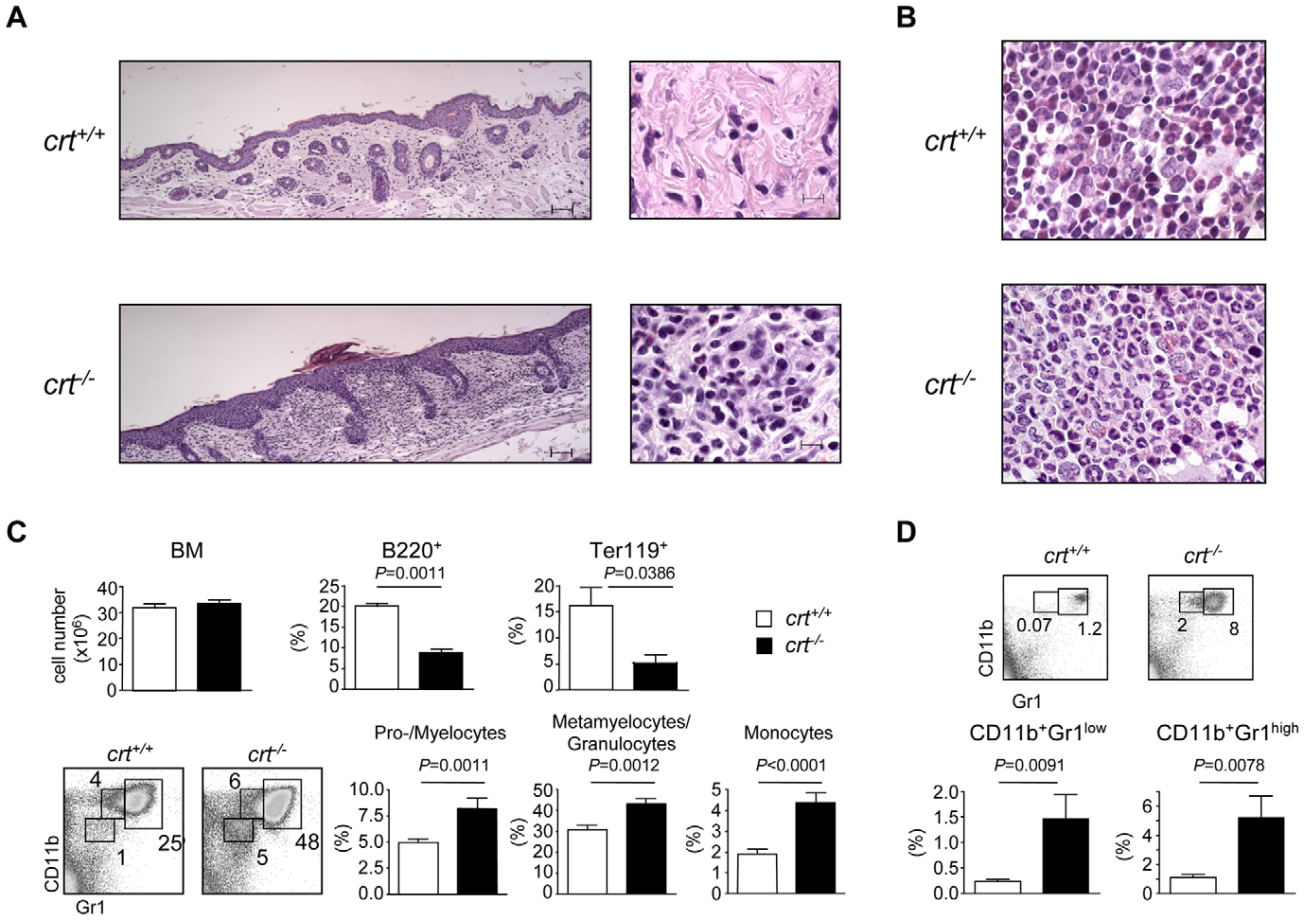
Histopathology and enhanced granulopoiesis in *crt^−/−^* FLC. (A) H&E stained sections show dermal granulocytic inflammatory infiltrate in the skin of *crt^−/−^* FLC at week 12 post-transplant, as opposed to *crt^+/+^* FLC, where inflammatory cells are absent (bar = 50 µm in left panels; bar = 10 µm in right panels). (B) Bone marrow sections of *crt^−/−^* FLC reveal a pronounced increase in myeloid precursors with left-shifted maturation and marked reduction of red cell precursors; *crt^+/+^* FLC retain the physiological myelo-erythroid ratio (original magnification, 63×). (C) Bone marrow cellularity with relative representations of B220^+^ (*crt^+/+^*, n = 35, *crt^−/−^*, n = 39) and Ter119^+^ (*crt^+/+^*, n = 8, *crt^−/−^*, n = 5) cells (top) at weeks 12–18 after reconstitution; dot plot analysis of BM cells stained with CD11b and Gr-1 antibodies and histograms with distribution of the indicated subsets (*crt^+/+^*, n = 16, *crt^−/−^*, n = 10). (D) Dot plot analysis of splenocytes stained with CD11b and Gr-1 antibodies and histograms with distribution of the indicated subsets (*crt^+/+^*, n = 11, *crt^−/−^*, n = 9). White bars: *crt^+/+^* FLC; black bars: *crt^−/−^* FLC.

*Crt^−/−^* T cells have an altered regulation of calcium signalling resulting in hyper responsiveness to TCR stimulation. In *crt^−/−^* FLC, this altered signalling results in a severe T cell mediated immunopathological condition starting at week 8 after reconstitution and characterized by blepharitis, alopecia and wasting syndrome [7]. The skin from these animals is characterized by inflammatory granulocytes mainly localized in superficial derma with focal infiltration of epidermis (Fig. 1a). In the BM, the myelo-erythroid ratio was altered by the presence of striking myeloid hyperplasia with left-shifted maturation (Fig. 1b). No differences in cell recovery from BM of both *crt^−/−^* and *crt^+/+^* FLC were detected, however, FACS analysis with specific lineage markers of the lymphoid, myeloid and erythroid compartment showed a significant increase in CD11b^+^Gr1^lo^ cells (promyelocytes/myelocytes), CD11b^+^Gr1^hi^ cells (metamyelocytes/granulocytes) [10], and CD11b^lo^Gr1^lo^ cells (mainly monocytes). Of note, we observed a decrease in B lymphoid (B220^+^) and erythroid (Ter119^+^) elements (Fig. 1c). Concomitantly, the increased granulopoiesis was correlated to the increase in both immature and mature granulocytes in the spleen of *crt^−/−^* FLC (Fig. 1d). These alterations were not observed in *crt^−/−^*/*cd3e^−/−^* double knock-out (KO) FLC, which do not develop inflammatory manifestations because of the absence of pathogenic T cells (Figure S1).

### Depletion of early stages of B cell development but not BM mature B cell compartment during inflammation

**Figure 2:**
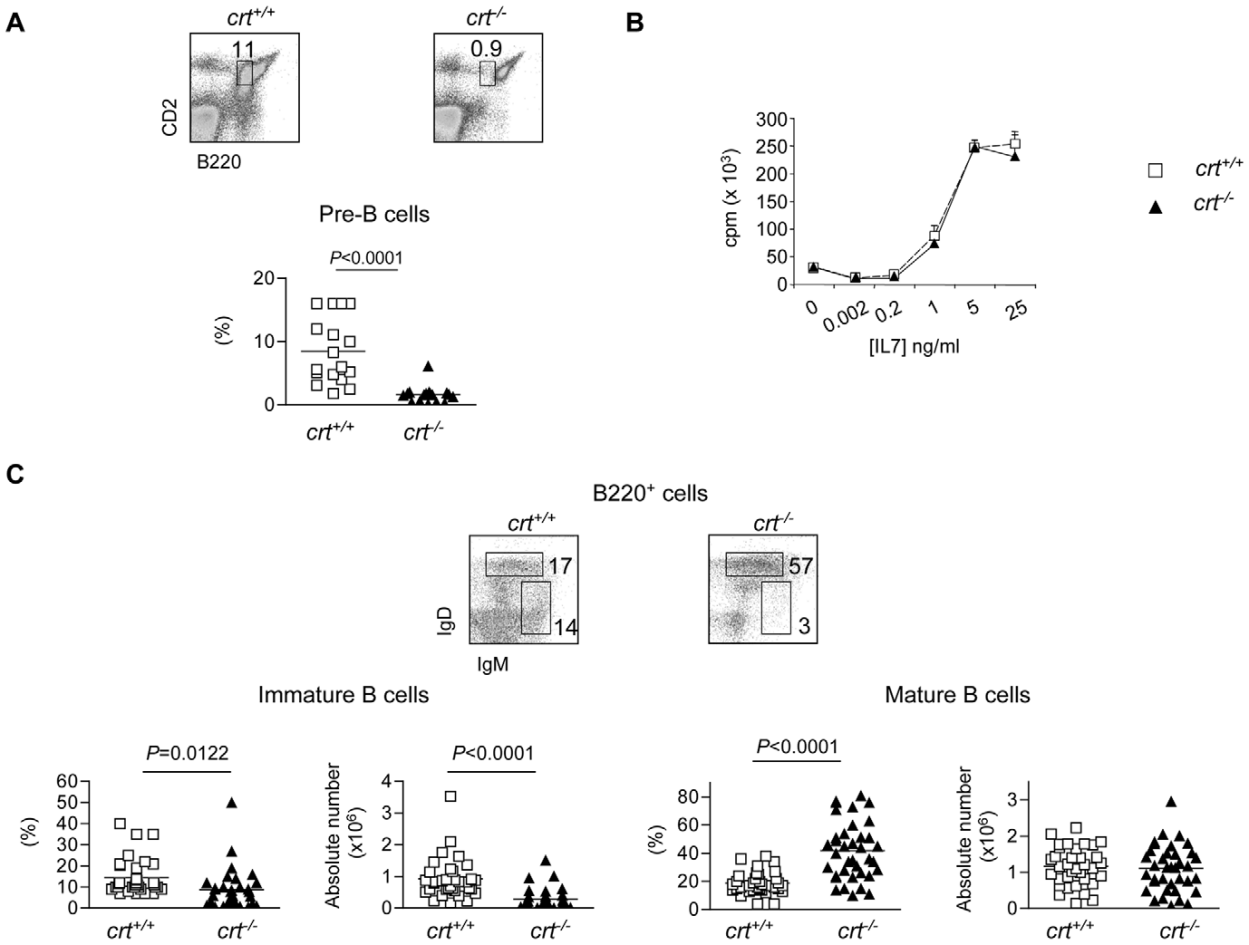
B cell progenitors but not mature B cells depletion in the BM of *crt^−/−^* FLC. (A) Dot plot analysis of BM cells from FLC stained with B220 and CD2 antibodies. The gate corresponding to pre-B cells used for the statistical analysis (below) is indicated. (B) ^3^H-thymidine incorporation to serial dilutions of recombinant IL-7 by sorted BM pro- and pre-B cells (see Results). (C) Dot plot analysis of electronically gated B220^+^ cells stained with IgM and IgD antibodies with statistical analysis of the respective subsets (below). Mice were analysed at weeks 12–18 after reconstitution.

To better define the B220^+^ cell defect with respect to B cell development in *crt^−/−^* FLC we first enumerated pre-B cells (B220^lo^CD2^+^) [2] (>98% CD19^+^) and immature B cells (B220^+^IgM^+^IgD^lo^). These analyses revealed a significant decrease of both cell subsets in *crt^−/−^* FLC (Fig. 2). Additional staining with CD43 and IgM antibodies showed the severe reduction of pre-B cells (Figure S2). Furthermore, we detected a dramatic depletion of CLP defined as lineage-negative c-kit^lo^, IL-7R^+^ cells (manuscript in preparation). In contrast, the relative representation, but not absolute number, of the mature B cell compartment (B220^+^IgM^lo/+^IgD^+^) was significantly increased (Fig. 2c).

To determine if the *crt^−/−^* B cell progenitors had an intrinsic functional impairment due to deletion of the *crt* gene, we evaluated the proliferative response of sorted B220^lo^CD43^lo/+^IgM^−^ cells comprising pro- and pre-B cells to IL-7 in vitro. Committed B cell precursors are known to depend on IL-7 for survival and expansion [11], [12]. Bone marrow samples were collected at week 4 and 5 after reconstitution, at which time mature T cells were not detected in the periphery and there were no signs of immunopathology in the *crt^−/−^* FLC. Similar percentages of pre-B cells defined by B220 and CD2 staining were observed in both *crt^−/−^* and *crt^+/+^* FLC at this time (*crt^−/−^*: 10.5±2.1; *crt^+/+^*: 9.7±0.9; n = 5), and other B cell developmental stages were not found to differ between these two mouse strains. Comparable ^3^H-thymidine incorporation by sorted B220^lo^CD43^lo/+^IgM^−^ *crt^−/−^* and *crt^+/+^* cells upon exposure to decreasing concentrations of IL-7 also was observed (Fig. 2b), indicating that *crt* deletion did not affect the expansion potential of early B cell progenitors. Moreover, in T cell depleted *crt^−/−^*/*cd3e^−/−^* double KO FLC that did not develop inflammation, the representations of the pre-, immature and mature B cell stages were indistinguishable in *crt^+/+^*/*cd3ε^−/−^* and *crt^−/−^*/*cd3ε^−/−^* FLC (Figure S1b,c). These data demonstrate that *crt* deletion in hematopoietic progenitors did not alter their B lymphopoietic potential.

**Figure 3:**
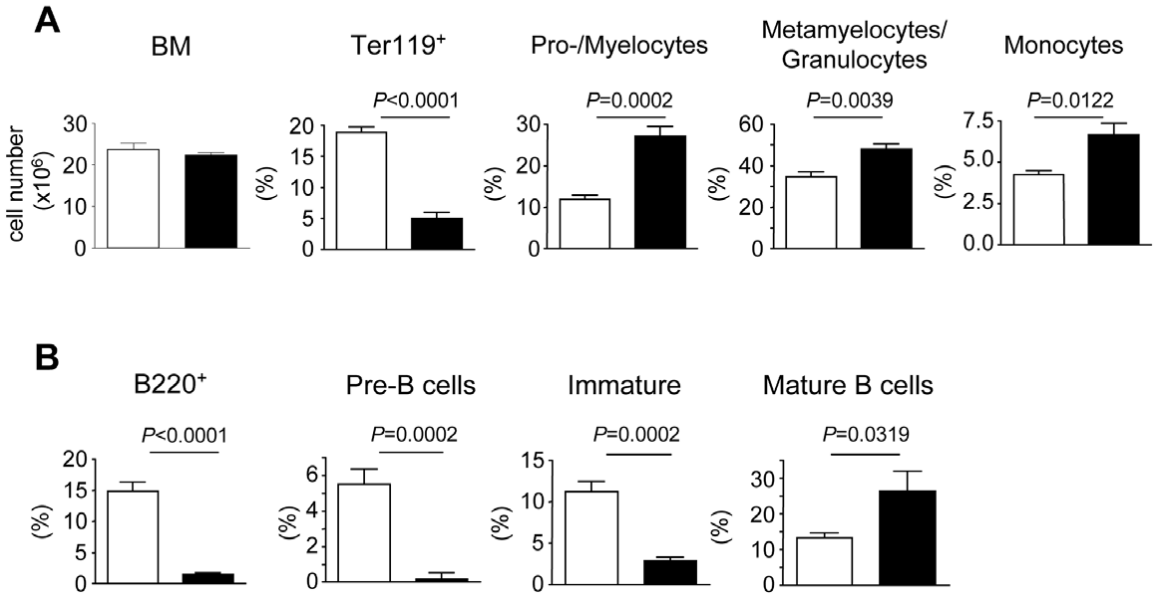
Erythroid, myeloid and B cell subsets in BM from IBD. (A) Bone marrow cellularity, percentages of Ter119^+^ cells and myelocytes subsets by CD11b and Gr-1 expression (see fig. 1 legend) in BM of mice with IBD, n = 5. (B) Statistical analysis of B cell subsets representation in BM of mice with IBD, n = 5. White bars: healthy; black bars: IBD. Mice were analysed 4 weeks after transfer of CD4^+^ cells.

To substantiate the alterations observed in *crt^−/−^* FLC as a general consequence of T cell mediated tissue inflammation, we used a murine model of IBD. We induced the disease by transferring CD4 T cells in the absence of the CD25^+^ T regulatory (T_reg_) subset into lymphopenic hosts. Indeed, a failure in the induction of regulatory T cell responses by gut microflora has been shown to cause severe mucosal inflammation by effector T cells that overproduce proinflammatory cytokines [8]. Naive CD4 T cells isolated from C57BL/6 mice were sorted as CD4^+^CD44^−^CD25^−^CD62L^+^ cells and injected into syngeneic *cd3ε^−/−^* mice. As a control, we used mice injected with sorted CD4 naïve cells together with sorted CD4^+^CD25^+^ cells [13] (see Methods). Mice were sacrificed at week 5 after cell transfer and IBD was assessed by weight loss (IBD mice: 15.5±0.9 g, n = 5; Control mice: 20.7±1.0 g, n = 5, *P* = 0.0001) and histological examination of the colon. As observed in *crt^−/−^* FLC, B220^+^ and Ter-119^+^ cells were significantly decreased whereas granulocytes and monocytes as well as their progenitors were significantly increased (Fig. 3a). The B220^+^ cells depletion was more dramatic than in *crt^−/−^* FLC, analogously to *crt^−/−^* FLC selectively affected pre- and immature B cells, whereas the percentage of mature B cells was significantly increased (Fig. 3b). These results demonstrate that depletion of the immature B cell pool and the relative preservation of BM mature B cells is a common feature of T cell mediated inflammation.

### Transcriptional regulation of CXCL12, SCF and IL-7 upon peripheral tissue inflammation

**Figure 4:**
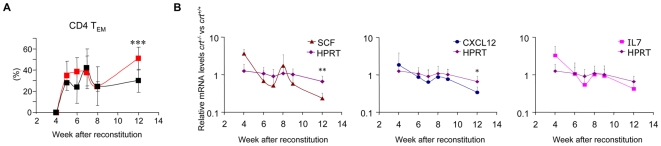
Kinetics of peripheral effector/memory T cells and transcripts in BM from FLC. (A) Percentages of peripheral CD4 effector/memory T cells in lymph nodes measured at different times following reconstitution of RAG/γ chain DKO mice with *crt^+/+^* and *crt^−/−^* fetal liver progenitors (mean ± SD of at least 5 mice). Black line, *crt^+/+^*; red line, *crt^−/−^*; *** = *P*<0.001. (B) Quantification by real time PCR of SCF, CXCL12, IL-7 and HPRT transcripts in BM at different time points after reconstitution (mean ± SD of at least 3 samples), ** = *P*<0.01 and * = *P*<0.05.

CXCL12 is released from BM stromal cells and is involved in retention of early B cell precursors within the BM microenvironment [14]. CXCL12 and SCF transcripts were downregulated in the BM upon immunization with incomplete Freund adjuvant (IFA), and this effect was correlated to the transient reduction of B cell progenitors [10]. We performed real-time PCR to quantify CXCL12, SCF, and IL-7 transcripts in *crt^−/−^* FLCs at different times after reconstitution. Kinetic analysis of these transcripts in *crt^−/−^* and *crt^+/+^* FLCs revealed the significant reduction of both CXCL12 and SCF, and the reduction of IL-7, albeit not to significance (Fig. 4b) in *crt^−/−^* FLC. This reduction in cytokine expression occurred concomitantly with progressing inflammation, as measured by the increase in lymph nodes effector/memory T cells (Fig. 4a). These results suggest that downregulation of both CXCL12, SCF and IL-7 could contribute to the reduction of B cell progenitors in the BM of mice during chronic inflammation. Accordingly, we detected significantly increased numbers of CD93^high^B220^low^IgM^−^ B cell progenitors in the spleen of mice with IBD with respect to healthy controls (Controls: 0.22×10^6^±0.04, n = 5; IBD: 1.76×10^6^±1.17, n = 5, *P* = 0.039).

### Depletion of marginal zone B cells in the spleen of *crt^−/−^* FLC and mice with IBD

**Figure 5:**
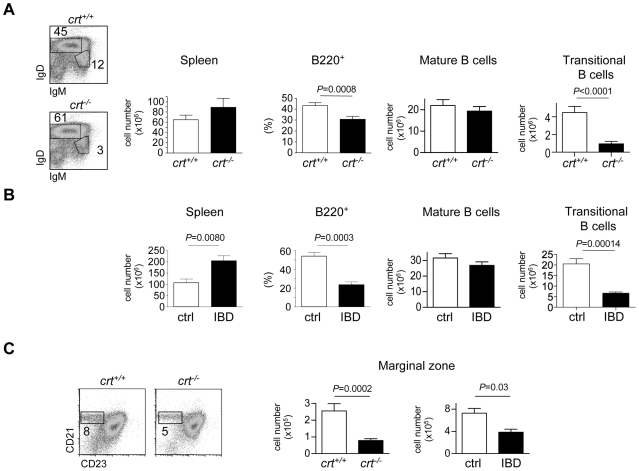
Depletion of MZ B cells in inflammation. (A) Dot plot analysis of electronically gated B220^+^ splenocytes stained with IgM and IgD specific antibodies in representative *crt^+/+^* and *crt^−/−^* FLC. Spleen cellularity, relative representation of B220^+^ cells, absolute numbers of mature FO B cells (IgD^+^IgM^lo/+^ gate in the dot plot) and transitional B cells (IgD^dull^IgM^+^ gate in the dot plot) in spleens from *crt^+/+^* (n = 35) and *crt^−/−^* (n = 31) FLC at weeks 12–18 after reconstitution. (B) The same analysis was performed on splenocytes from healthy mice (ctrl, n = 5) or mice with IBD (IBD, n = 5) 4 weeks after adoptive cell transfer. (C) Representative dot plots on B220^+^ cells with MZ B cells gate (left graphs), statistical analyses of absolute numbers of MZ B cells in *crt^+/+^* and *crt^−/−^* FLC at weeks 12–18 after transplant (*crt^+/+^*, n = 24, *crt^−/−^*, n = 30) (middle graph) and healthy mice (ctrl, n = 5) or mice with IBD (IBD, n = 5) 4 weeks after adoptive cell transfer (right graph).

Spleen cellularity was increased in chronic inflammation but the percentage of B220^+^ cells was significantly reduced. Mature FO B cells (IgM^lo/+^IgD^+^) were not significantly altered, whereas transitional B cells [15] were significantly diminished in both *crt^−/−^* FLC and mice with IBD (Fig. 5a,b), in line with a reduced BM output of immature B cells. To evaluate the impact of chronic inflammation on marginal zone B cells representation, we stained splenocytes from *crt^−/−^* FLC and mice with IBD with B220 together with CD21 and CD23 specific antibodies. Figure 5c shows the significant reduction of CD21^high^ CD23^lo^ MZ B cells [16] in both inflammatory conditions.

### Transcriptional profiles of mature B cell subsets and responsiveness to TLR9 ligand

**Figure 6:**
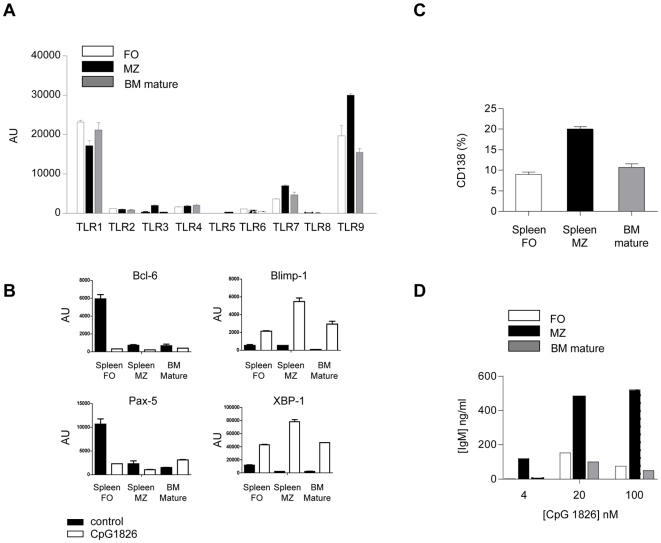
TLRs expression and response of BM mature, FO and MZ B cells to CpG 1826. (A) Representative experiment showing TLRs expression by quantitative real-time PCR on sorted resting B cell subsets. AU: arbitrary unit. Mean values of triplicates ± SD from a single experiment are shown. (B) Expression of Bcl6, Blimp-1, Pax-5 and XBP-1 transcription factors by real-time PCR on the indicated B cell subsets either non stimulated (black bars) or after 48 h of stimulation with CpG at 100 nM (white bars). AU: arbitrary unit. Mean values of duplicates ± SD from a single experiment are shown. (C) Percentages of CD138^+^ B cells after 4 day stimulation of the indicated B cell subsets with CpG 1826 at 100 nM. (D) IgM secretion by the indicated B cell subsets after 4 day stimulation with CpG 1826 at different doses.

The transcription of TLRs differs quantitatively but not qualitatively in FO and MZ B cells [17]. The analysis of MZ versus BM mature B cells in three different experiments revealed increases, albeit not reaching statistical significance, in TLR7 and 9 transcripts, whereas TLR3 was exclusively expressed in MZ B cells. Figure 6a shows results obtained in one representative experiment. In vitro stimulation of purified FO, MZ and BM mature B cells with TLR9 ligand CpG 1826 revealed the more efficient transition of MZ B cells to Ig secreting cells (ISCs), as measured by CD138 expression (Fig. 6c) and Ig secretion (Fig. 6d). Transcriptional regulation of Pax5, Bcl-6, Blimp-1 and XBP-1 controls differentiation of B cells to plasma cells. Reciprocal expression of Bcl-6 and Blimp-1 ensures the differential fate of activated B cells either to enter the germinal center or differentiate into ISC, respectively [18]. In resting conditions mature BM B cells displayed low levels of Bcl-6 and Pax-5 transcripts, similar to MZ B cells. In contrast, FO B cells displayed high levels of Bcl-6 and Pax-5 transcripts. After CpG stimulation however, mature BM B cells displayed less robust upregulation of Blimp-1 and XBP-1, similarly to FO B cells (Fig. 6b). In spite of some different Pax-5 transcript levels in MZ versus FO B cells observed by Genestier et al. [17], analogous downregulation of Pax-5 in MZ B cells upon TLR9 stimulation was observed. These results suggest that MZ B cells differentiate more readily into plasma cells by stimulation with TLR ligands generated by tissue damage during T cell mediated inflammation, thereby leading to their exhaustion in concomitance with depressed B lymphopoiesis.

### Role of macrophage migration inhibitory factor (MIF) in the preservation of BM mature B cell pool upon peripheral tissue inflammation

**Figure 7:**
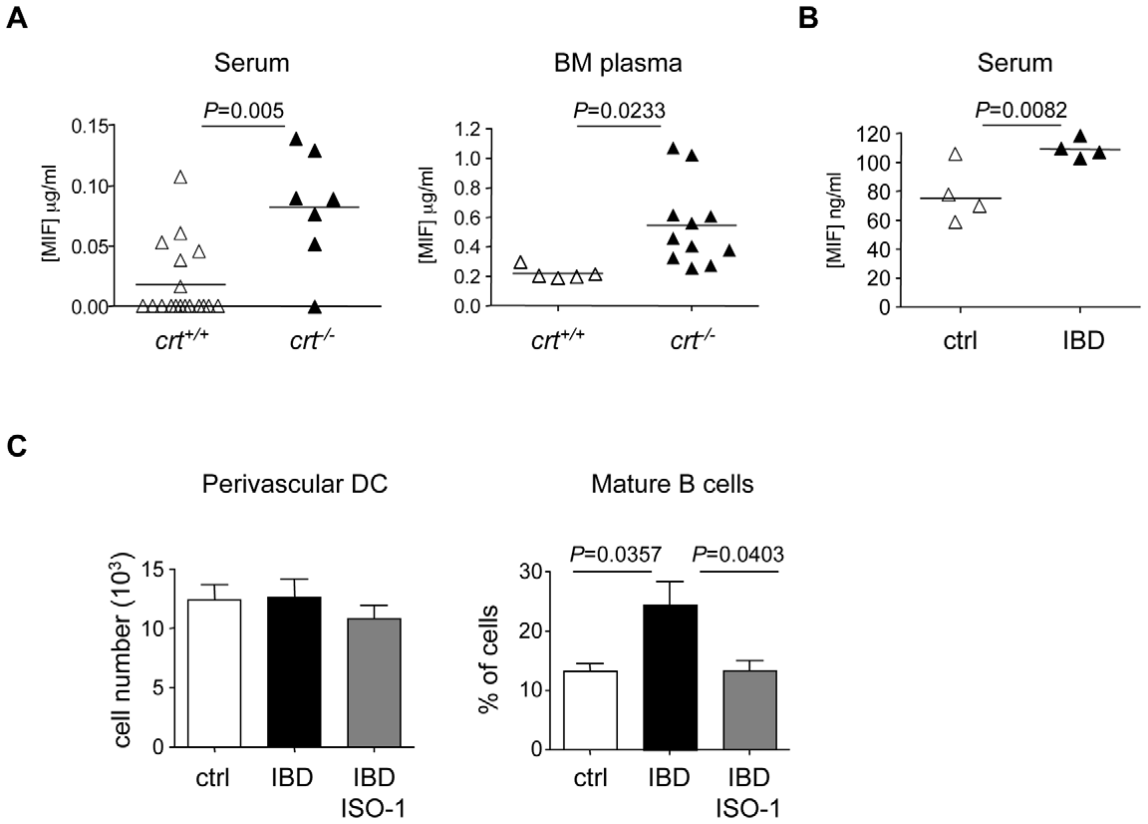
Increased MIF concentrations in inflammation and selective depletion of BM mature B cells by ISO-1. (A) Serum and BM plasma concentrations of MIF in FLC. (B) Serum concentrations of MIF in healthy mice (ctrl) or mice with IBD (IBD). (C) Absolute numbers of F4/80^+^CD103^+^ perivascular DC identified as CD11c^high^MHCII^high^ and percentages of BM mature (IgD^+^IgM^lo/+^) cells in gated B220^+^ cells in healthy mice (ctrl) or mice with IBD either mock treated (IBD) or treated with ISO-1 (IBD/ISO-1).

Recently, MIF produced by BM perivascular dendritic cells was shown to be crucially involved in the survival of BM mature B cells [9]. We measured serum and BM plasma levels of MIF at week 12 after reconstitution in *crt^+/+^* and *crt^−/−^* FLC. Significant increases in MIF concentrations were detected in both serum and BM plasma from *crt^−/−^* FLC (Fig. 7a), thus suggesting that increased MIF secretion might ensure the maintenance of the BM mature B cell pool during inflammation. Analogously, mice with IBD displayed increased serum levels of MIF with respect to healthy controls (Fig. 7b).

To establish a causal relationship between increased MIF and BM mature B cells preservation during inflammation, we treated mice with IBD for six days by intravenous administration of ISO-1. ISO-1 is a non-toxic inhibitor of MIF that binds to bioactive MIF at a catalitically active tautomerase site [19], [20]. This treatment did not modify the representation of F4/80^+^CD103^+^ perivascular DC identified by staining with CD11c and MHC class II specific antibodies. However, mature B cells were significantly reduced in treated mice (Fig. 7c, Figure S3), indicating that MIF was mediating the preservation of BM mature B cell pool during chronic inflammation.

## Discussion

Newly developed B cells migrate from the BM to the spleen where they primarily mature into recirculating FO B cells and also differentiate into non recirculating MZ B cells, which are strategically located to respond to blood-borne pathogens in a T-independent fashion [3]. Recently, mature B cells residing in a perivascular niche in the BM also were characterized as responsive to blood-borne pathogens in a T-independent fashion, analogously to MZ B cells [4]. Chronic inflammation with severe tissue damage skews hematopoiesis toward the granulocyte/monocyte lineage with concomitant reduction of erythroid Ter-119^+^ cells and depression of B lymphopoiesis. Both megacaryocyte/erythrocyte progenitors (MEPs) and common lymphoid progenitors (CLPs) were significantly reduced in both *crt^−/−^* FLCs and mice with IBD, whereas granulocyte/monocyte progenitors (GMPs) were significantly increased (manuscript in preparation). In addition, we detected immature B cell progenitors in the spleen of mice with IBD concomitantly with downregulation of CXCL12, as observed in acute inflammation [6]. Our study revealed that inflammatory tissue damage affects the MZ B cell population. Inflammation might represent a chronic source of TLR agonism and it has been noted that TLR agonists promote differentiation to ISC of MZ but not FO B cells. This might explain the preferential depletion of the former B cell population during chronic inflammation, in an analogous fashion to what observed during infection with different T-independent pathogens [21], [22]. Mature BM B cells share transcriptional profiles with MZ B cells and differentiate into Ig secreting cells by stimulation with TLR agonists, albeit with an efficiency comparable to FO rather than MZ B cells. Both BM mature and MZ B cells have a long half-life [23], however the reduced TLR responsiveness and the recirculating nature of the BM mature B cell pool with possible replenishment by selection from the FO B cell pool may explain the differential sensitivity of these two B cell compartments to chronic tissue damage.

Since a minor fraction of mature B cells differentiate in the BM from transitional B cells without any requirement for the spleen or other secondary lymphoid organs [24], it is also possible that the perisinusoidal niche where this compartment homes to is less affected by increased granulopoiesis. Some measure of B cell development thus may continue to take place at this site. A topographical analysis of B cell distribution in the rat BM has shown that pre-B but not mature B cells were selectively enriched in the subendosteal area [25]. Ueda et al. has suggested that granulopoiesis and lymphopoiesis share the same developmental niche and compete for the same developmental resources [10]. The sparing of the B cell developmental potential of the perisinusoidal niche would ensure “emergency” B lymphopoiesis and protection against blood-borne pathogens during inflammatory tissue damage.

A subset of bone marrow-resident dendritic cells organized in perivascular clusters was shown to provide crucial survival signals for mature recirculating B cells through the production of MIF [9]. Indeed, MIF was shown to induce B cell survival by activation of the CD74-CD44 receptor complex [26]. Serum and BM plasma levels of MIF were increased upon T cell mediated inflammation. We used the specific MIF inhibitor ISO-1 [19] to show that the selective preservation of the mature BM B cell subset during experimentally-induced IBD was dependent on MIF biological activity. Altogether, the data presented in this study allow for a model whereby T cell mediated inflammation could impact on the MZ B cell potential to generate T-independent responses against blood-borne pathogens. This possible vulnerability is counteracted by the MIF-mediated preservation of the recirculating mature B cell pool in the BM, thus ensuring serological responsiveness to blood-borne microbes.

One possible drawback in the dominant responsiveness to blood-borne antigens by BM mature, recirculating B cells may originate from the distinct repertoire of this subset with respect to MZ B cells. Indeed, the selection of cells lacking N regions and with shorter CDR3s in the immunoglobulin repertoire of MZ B cells but not BM mature B cells was hypothesized to confer to MZ B cells a reduced potential for harmful autoreactivity [27]. Accordingly, TdT deficiency was shown to reduce autoimmune disease incidence and severity [28], [29]. The preferential responsiveness of BM recirculating B cells to blood-borne pathogens thus may impose an autoimmune hazard to the organism during chronic inflammation.

## Materials and Methods

### Ethics Statement

Experiments were approved by the “Dipartimento della Sanità e della Socialità” with authorizations 8/2007, 10/2007 and 01/2009.

### Mice

*Crt^+/−^* (H-2^b^) mice [30], BALB/c RAG/γ chain DKO (H-2^d^) mice obtained from Dr. Mamoru Ito (Central Institute for Experimental Animals, Miyamae, Kawasaki, Japan) and *cd3ε^−/−^* mice from Jackson Laboratory were bred and treated in accordance with the Swiss Federal Veterinary Office guidelines. FLC in RAG/γ chain DKO mice with *crt^+/+^* and *crt^−/−^* progenitors were generated as described [7]. Briefly, sublethally irradiated mice were injected i.v. with 2×10^6^ genotyped fetal liver cells in 200 µl PBS. Similarly, FLC with *crt^−/−^/cd3ε^−/−^* and *crt^+/+^/cd3ε^−/−^* progenitors were generated by breeding *crt^+/−^/cd3ε^−/−^* mice.

### Histochemistry

The mice were dissected and tissues were immediately fixed in buffered formalin. A multi-organ microscopic analysis, including hematopoietic organs, was carried out. Bone marrow was obtained from femurs, fixed, and decalcified in EDTA-based solution. Each specimen was paraffin-embedded, cut into 4 µm-thick sections and stained with Hematoxylin & Eosin (H&E) and Giemsa. Individual specimens were independently analysed by two of us (M.P and F.S), without knowledge of mice genotype.

### Flow cytometry

For flow cytometry analyses, mAbs conjugated with either biotin, FITC, PE, CyChrome, PerCP or APC against the following antigens were used: CD8α (53-6.7), CD4 (L3T4), CD62L (MEL-14), CD44 (IM7), CD25 (PC61.5), Gr-1 (RB6-8C5), CD2 (RM2-5), TER-119, CD11b (M1/70), CD19 (6D5), CD21 (8D9), CD23 (B3B4), CD43 (R2/60), CD49b (DX5) and MHC class II (M5/114.15.2) (eBioscence); CD45R/B220 (RA3-6B2), IgM (II/41), IgD (11.26c.2a), and APC-Cy7-streptavidin (BD Bioscience). All samples were acquired with a FACScalibur or FACSCanto (Becton Dickinson Immunocytometry Sistems) and analyzed with FlowJo software (Treestar Inc.).

### Pro-, pre-B cell proliferation assay

B cells were isolated from BM by negative selection with immunomagnetic beads followed by cells sorting. Cell suspensions were incubated with biotin-conjugated antibodies (Ter119, panNK, CD11b, Gr-1, CD4 and CD8) (eBioscence) and B cell enriched by removing biotin-labeled cells with streptavidin conjugated magnetic beads (Miltenyi Biotec). Pro/pre B (B220^+^IgM^−^CD43^+^) cells were sorted at FACSAria (Becton Dickinson), plated at 2×10^4^ cells/well, and cultured in presence of rIL7 in 96 well plates. At day 4 [^3^H]-thymidine (2 µCi/well) was added and the plates were harvested after 16 h incubation.

### Induction of inflammatory bowel disease

For the induction of IBD, T cells were isolated from the peripheral lymph nodes and spleen of C57BL/6 mice by negative selection with immunomagnetic beads followed by cells sorting. Cell suspensions were incubated with biotin-conjugated antibodies (Ter119, panNK, CD11b, Gr-1 and CD19) (eBioscence) and T cells enriched by removing biotin-labeled cells with streptavidin conjugated magnetic beads (Miltenyi biotec). CD4^+^ naïve (CD4^+^CD44^−^CD25^−^CD62L^+^) and regulatory (CD4^+^ CD25^+^) T cell subsets were sorted at FACSAria (Becton Dickinson). *Cd3ε^−/−^* mice were i.v. injected with 0.4×10^5^ CD4^+^ naïve T cells. As a control, mice were injected with CD4 naïve cells together with 0.2×10^5^ CD4^+^CD25^+^ T_reg_ cells. Mice were weighted weekly and were sacrificed at week 5 after cell transfer, when IBD was diagnosed by severe diarrhea and weight loss. For ISO-1 (Calbiochem) treatment, mice were injected 4 weeks after cell transfer for 6 days with 20 mg/kg of ISO-1 intravenously. Control mice were injected with carrier alone.

### Quantification of mRNA levels

mRNA from 0.2×10^6^ BM cells was precipitated in Trizol (Invitrogen) and reverse transcribed (M-MLV, Invitrogen). Quantitative PCR amplifications of cDNA were performed in an ABI PRISM 7700 Sequence Detector (PE Applied Biosystem) with SYBR Green PCR core reagents (Applied Biosystems) and primers specific for HPRT, GAPDH, SCF, CXCL12 or IL7 transcripts. The following standard amplification parameters for the quantitative PCR were used: initial denaturation at 94°C for 10 min; amplification cycle; denaturation at 94°C for 45 s; and anneal/extension at 64°C for 45 s. The relative gene expression levels were calculated by the comparative C_T_ (threshold cycle) method recommended by the manufacturer (Applied Biosystems) normalized to GAPDH message in the same sample, as described [10]. In brief, DC_T_ values were determined by subtracting C_T (GAPDH)_ from C_T (target)_. Expression levels relative to GAPDH were defined as: 2^−DCT^. Expression data were standardized to averaged, homologous message in *crt^+/+^* BM cells. Levels of a second housekeeping gene, HPRT, were used as control for nonspecific changes in message levels. The following primers were used [10], [31]:

HPRT forward 5′-GCTGGTGAAAAGGACCTCT-3′

  reverse 5′-CACAGGACTAGAACACCTGC-3′

GAPDH forward 5′-AACTTTGGCATTGTGGAAGG-3′

  reverse 5′-ACACATTGGGGGTAGGAACA-3′

SCF forward 5′-CGGGAATCCTGTGACTGATAA-3′

  reverse 5′-GGCCTCTTCGGAGATTCTTT-3′

CXCL12 forward 5′-GTCCTCTTGCTGTCCAGCTC-3′

  reverse 5′-TAATTTCGGGTCAATGCACA-3′

IL-7 forward 5′-TGGAATTCCTCCACTGATCC-3′

  reverse 5′-ACCAGTGTTTGTGTGCCTTG-3′

### TLRs expression and responsiveness of BM mature, FO and MZ B cells to CpG 1826

TLRs expression was evaluated on sorted, non-stimulated B cells. B cells were isolated from spleen and BM by positive selection with immunomagnetic beads specific for B220 (Miltenyi Biotec). Then, MZ (B220^+^CD21^high^CD23^lo^), FO (B220^+^CD21^+^CD23^+^) and BM mature (B220^+^IgM^+^IgD^+^) B cells were sorted at FACSAria (Beckton Dickinson). For quantitative assessment of relative mRNA levels, total RNA was prepared from FACS-sorted population using Trizol LS reagent according to the manufacturer's instructions. RNA then was reverse transcribed using the M-MLV RT reverse transcription kit with random primers (Invitrogen). The relative mRNA levels were determined by real-time PCR on an ABI PRISM 7900 HT sequence detector (Applied Biosystems) using the assay on demand product for TLRs (TLR1 Mm00441868_s1, TLR2 Mm00442346_m1, TLR3 Mm01207403_m1, TLR4 Mm00445273_m1, TLR5 Mm00546288_s1, TLR6 Mm02529782_s1, TLR7 Mm00446590_m1, TLR8 Mm01157262_m1, TLR9 Mm00446193_m1), Blimp1 (Mm01187285_m1), XBP1 (Mm00457360_g1), Bcl6 (Mm00477635_m1) and Pax5 (Mm01345231_m1).

To analyse B cell responsiveness to CpG 1826, B cell subsets were stimulated at 3×10^4^ cells/well with CpG 1826 at 4, 20 and 100 nM. At day 4, immunoglobulin production was evaluated in the supernatant with a standard ELISA assay. Briefly, 96 flat bottom plates (Greiner) were coated with isotype specific goat anti-mouse IgM antibodies (Southern Biotechnologies) diluted in Na_2_HPO_4_ 0,2 M pH 9.6 and incubated overnight at 4°C. Plates were washed and blocked with PBS with 10% FBS for two hours at room temperature. After washing, serial dilutions of culture supernatants were added and incubated for two hours at room temperature. Plates were washed again and alkaline phosphatase-conjugated goat anti-mouse IgM was added and incubated for two hours at room temperature. The reaction was developed with Sigma 104 substrate (Sigma). Transcription factors expression was analyzed on B cell subsets after 48 hour stimulation with CpG 1826.

### Preparation of BM plasma

BM plasma was prepared by flushing femurs and tibias from each mouse with 500 µl RPMI 1640 containing 0.5% BSA and 10 mM Hepes. After first centrifugation at 1500 rpm to collect BM cells, debris were removed by repeated (2×) centrifugation at 2,000 rpm for 10 min.

### MIF quantification by ELISA

BM plasma and blood serum were assayed directly for MIF content by a sandwich ELISA. Briefly, 96-well microtiter plates (Greiner bio-one) were coated with the anti-mouse MIF mAb (15 µg/ml in PBS), washed, and blocked with Superblock (Pierce). Fifty-µl aliquots of each sample were added to wells and incubated overnight at 4°C. The wells were washed and incubated with rabbit polyclonal anti-MIF serum (1 µg/ml) for 2 h at room temperature. This was followed by the addition of an anti-goat IgG-HRP (Santa Cruz Biotechnology), and the antibody complexes were quantified after the addition of TMB (3-3′-5-5′ tetrametilbenzidin) and H_2_O_2_ (R&D System). The MIF concentrations were calculated by extrapolation from a sigmoidal quadratic standard curve using mouse rMIF.

### Statistical analysis

Student's paired *t* test was used to determine the significance of differences between mean values. Data are reported as mean ± SEM except where indicated. Values of *P*<0.05 were considered significant.

## Supporting Information

Figure S1 Unaltered BM lymphoid and myeloid lineage representation in *crt^−/−^/cd3e^−/−^* double KO FLC. (A) Statistical analyses of the indicated subsets in the BM of *crt^+/+^/cd3e^−/−^* and *crt^−/−^/cd3e^−/−^* FLC at week 12 from reconstitution. (B) Dot plot analyses of BM cells from *crt^+/+^/cd3e^−/−^* and *crt^−/−^/cd3e^−/−^* FLC stained with B220 and CD2, and statistical analysis of the pre-B cell subset (below). (C) Dot plot analysis of electronically gated B220^+^ BM cells stained with IgM and IgD antibodies and histogram distribution of the indicated subsets (below), n = 4.
(0.66 MB TIF)

Figure S2 Depletion of pre-B cells in the BM of *crt^−/−^* FLC. Statistical analysis of pre-B cells in the BM from FLC stained with B220, CD43 and IgM specific antibodies.
(0.10 MB TIF)

Figure S3 Reduction of BM mature B cells in IBD by ISO-1. Statistical analysis of absolute numbers of mature (IgD^+^IgM^lo/+^ in gated B220^+^ cells) B cells isolated from the BM of healthy controls (Treg), mice with IBD either mock treated (IBD) or treated with ISO-1 (IBD/ISO-1).
(0.08 MB TIF)
