# Supplementary figures and images for: Selective Preservation of Bone Marrow Mature Recirculating but Not Marginal Zone B Cells in Murine Models of Chronic Inflammation
Source: PLoS One. 2010 Jun 22;5(6):e11262. doi: 10.1371/journal.pone.0011262 (PMC2889832; doi:10.1371/journal.pone.0011262)

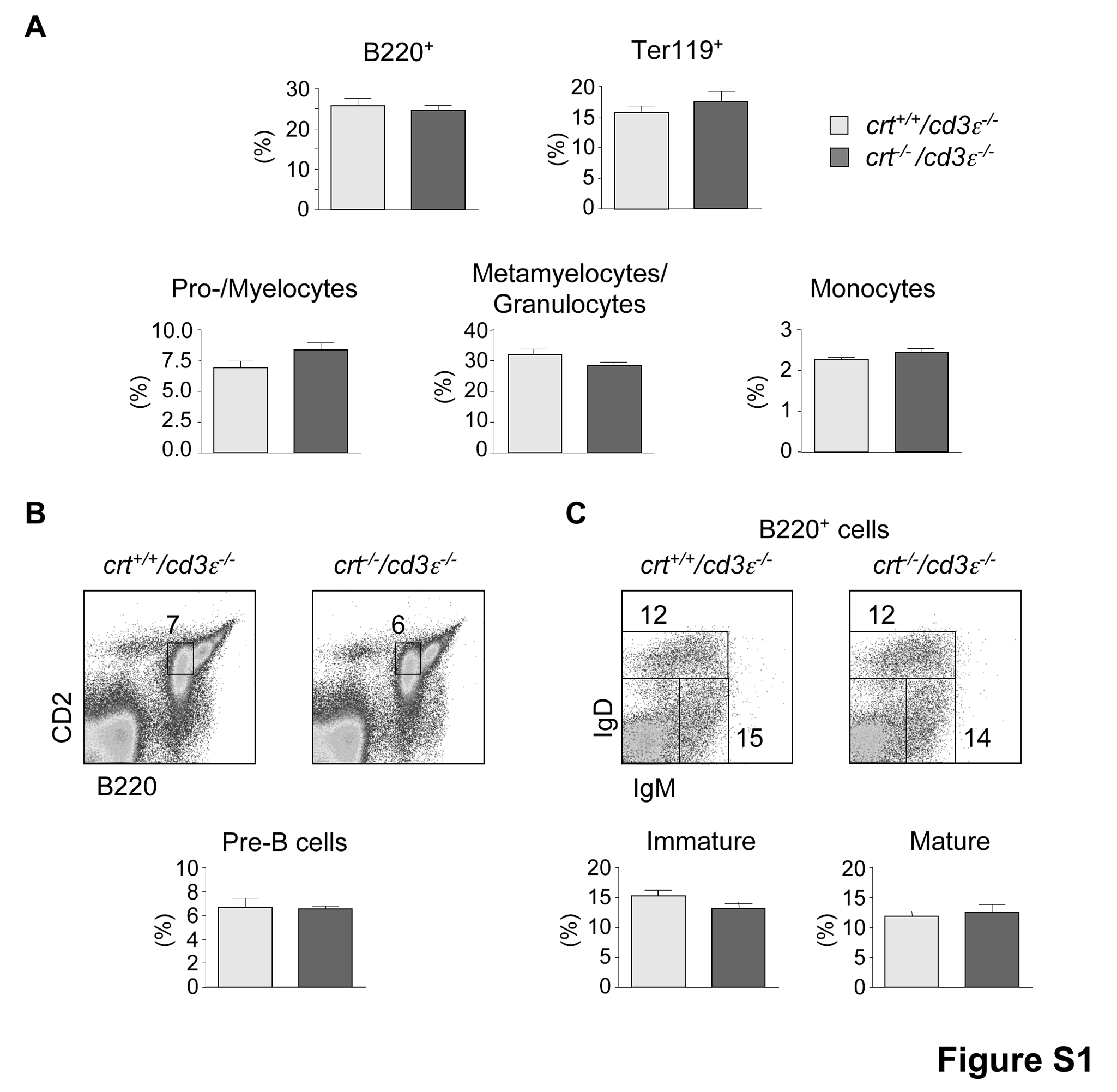

Supplement: Figure S1 — Unaltered BM lymphoid and myeloid lineage representation in crt−/−/cd3e−/− double KO FLC. (A) Statistical analyses of the indicated subsets in the BM of crt+/+/cd3e−/− and crt−/−/cd3e−/− FLC at week 12 from reconstitution. (B) Dot plot analyses of BM cells from crt+/+/cd3e−/− and crt−/−/cd3e−/− FLC stained with B220 and CD2, and statistical analysis of the pre-B cell subset (below). (C) Dot plot analysis of electronically gated B220+ BM cells stained with IgM and IgD antibodies and histogram distribution of the indicated subsets (below), n = 4. (0.66 MB TIF) [file pone.0011262.s001.tif]

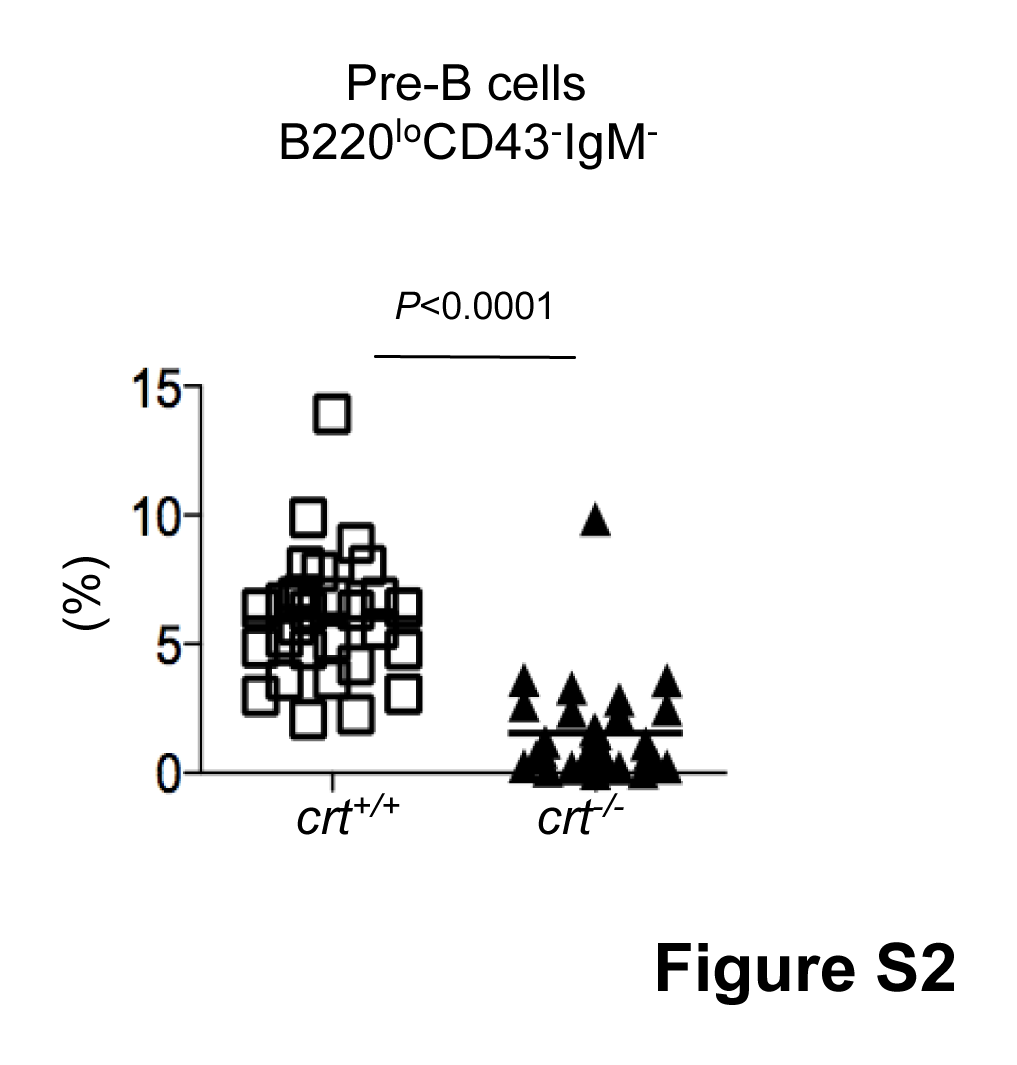

Supplement: Figure S2 — Depletion of pre-B cells in the BM of crt−/− FLC. Statistical analysis of pre-B cells in the BM from FLC stained with B220, CD43 and IgM specific antibodies. (0.10 MB TIF) [file pone.0011262.s002.tif]

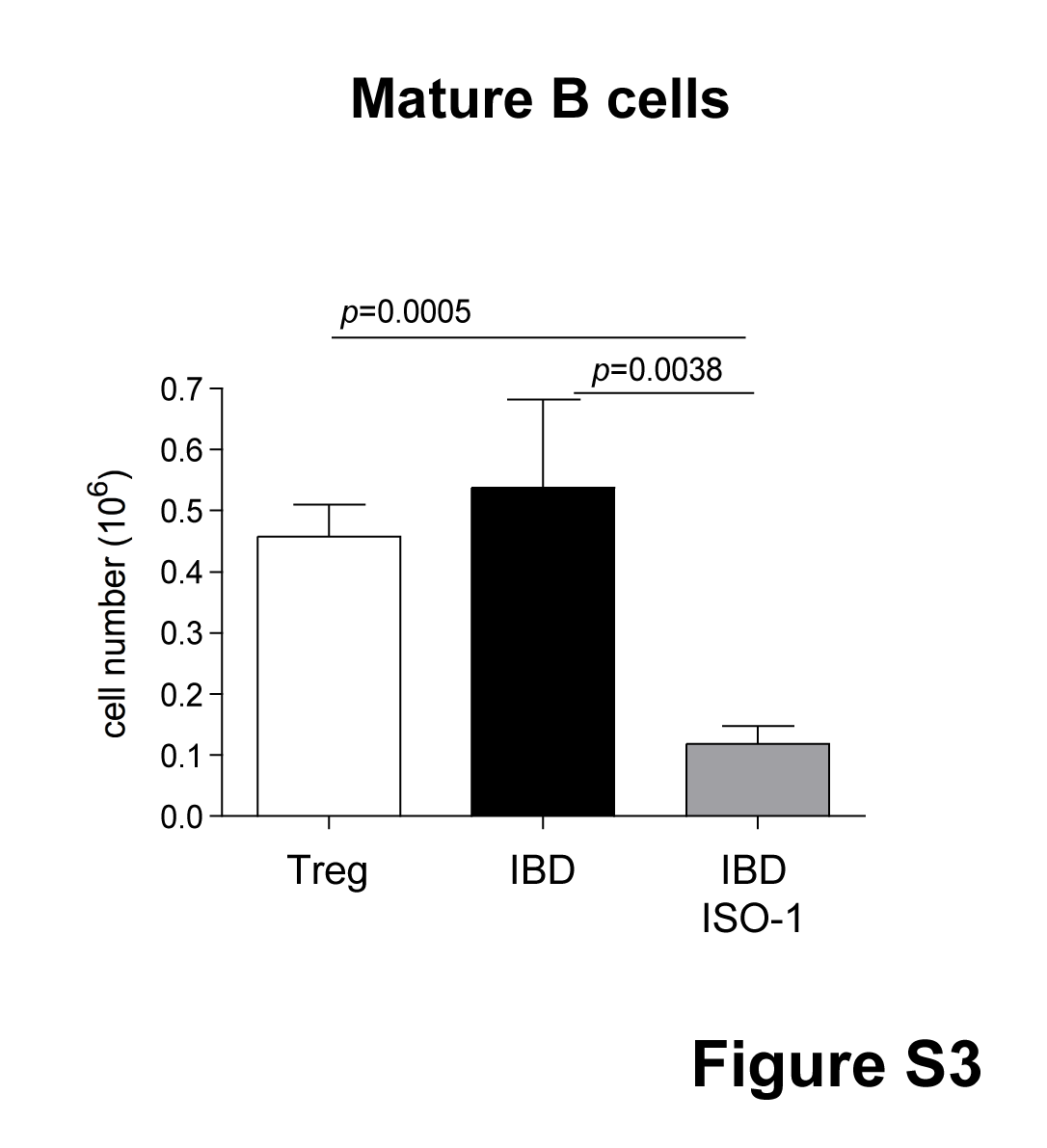

Supplement: Figure S3 — Reduction of BM mature B cells in IBD by ISO-1. Statistical analysis of absolute numbers of mature (IgD+IgMlo/+ in gated B220+ cells) B cells isolated from the BM of healthy controls (Treg), mice with IBD either mock treated (IBD) or treated with ISO-1 (IBD/ISO-1). (0.08 MB TIF) [file pone.0011262.s003.tif]
